# Histology of the Teeth

**Published:** 1879-03

**Authors:** J. H. Siddall

**Affiliations:** Canton, Ohio


### THE DENTAL REGISTER.

VOL. XXXIII.] MARCH, 1879. No 3.

### HISTOLOGY OF THE TEETH.

BY J. H. SIDDALL, CANTON, OHIO.

The ultimate constitutents of the teeth, in respect to organism, were unknown to antiquity and even to the middle ages, although Galen and Fox speak of the homogeneous and heterogeneous parts of the body, yet the minuter structures were veiled from them as well as others of their time. But each succeeding decade, since the application of the simple microscope to medicine, by Marcello Malpighi, (born 1628, died 1694), has, by its scientific workers, delved into the labyrinths of Nature's hidden mysteries, and converted theory into fact, until in the researches of Hassall, of 1851, Kolliker, of 1854, and Beale, of 1878, we have inherited volumes of priceless matter, as a result of microscopical investigations, and yet the goal is not reached, for much remains to be demonstrated, even of odontology.

It is not my purpose, in this dissertation, to give all the developmental steps of the tooth's formation, and will begin with the observation that its true blastema is the bioplasm, or living, germinal matter of the blood. The corpuscles, in their protein organism, having served their important function of conveying oxygen from the lungs and returning carbon from the capillaries, suffer dissolution and by their disintegration increase the quantity of fluid fibrin in the blood, which, being by exosmosis exhaled, becomes the true bioplasm of the fabric. Not that fibrin is germinal matter or bioplasm but of the living germinal matter of the body, fibrin is the main constituent. But that I may be fully understood in the use of the term bioplasm, I will quote from the fourth edition of Dr. Beale: "All organism, vegetable as well as animal, in the earliest stage of development, consists of a minute mass of clear, transparent, structureless, living matter, possessing formative power of the most remarkable kind, capable of undergoing conversion into matter which did not before exist—this is bioplasm,"—not cells or nucleus, for this distinguished author disclaims the old cell doctrine, and as a subsequent development of formed material from this bioplastic formation we find the papillæ of the tooth as one result! Placing its future well developed pulp, stripped of its cortical structure, upon the field of the microscope, we find what would seem, and what anatomists have falsely assumed, a single nerve and artery entering each root, but when passed under the compressorium, we find a fasciculus of from ten to fifteen or more well defined branches, these pass vertically into the pulp, and after forming a circuit, terminate in loops upon its periphery. Ends of nerves are spoken of by Kolliker, and I believe others, but I have failed to find an end or an abrupt turn in any instance, save the fusiform fibres that now and then are seen as sporules of the cells in the larger organs of the central system. This fasiculus is a continuation of the superior and inferior dental and is from the casserian ganglion of the trigeminus, and are consequently nerves of the central system. They consist of three distinctive parts—a neurilemma or covering, a whitish colloid mass, (medullary matter) in the center of which is a soft elastic fibre. The neurilemma is extremely delicate, transparent and elastic, and by Kolliker, claimed to be a structureless membrane, but indeed I know of no more highly structural organism than this. I have a specimen taken from one of the fasciuli of the spinal cord, which under a power of five hundred shows a most remarkable net work of elastic fibres and structural arıangement. And from its continuity of fibres and numerous loops of nerves, there remains not a doubt in my mind that the neurilemma is the main medium by which sensations are conveyed to the brain. In fact, as this is not irrelevent to my subject, I shall offer such proofs as have been suggested to my mind in the course of my investigations:

I. It is asserted by reputable microscopists that the spinal accessory nerves, although sensitive, have no cells. If this be true, from what other source do they receive their impressions? It can not be from their oil globules! ! for fibres do not exist in the bioplastic mass of medullary substance, independent of cells! Again I have made very many observations of both the white and gray substance of many of the larger trunks and ganglion, and I find but little difference in their appearance, except that there are more cells in the cervical part of the spinal column, the ganglion and the cardiac plexus, and in no instance is there to be found anything like connective tissues or loops of nerves. The nearest approximation, being the presence of an occasional fusiform fibre, which appears to be continuous with a cell, but which divides into forks, which are sufficiently isolated to render them powerless as contributors of nervous sensation. From three to ten small branches of the dental arteries enter each pulp and form a plexus of capillaries from which the veins arise. There is nothing in the circulation that I know of that demands further description.

The limitary or basement membrane that envelopes this vascular mass, bears the scars of many awkward and scientific thrusts. I say scientific because I have heard it stated within this room, that to restore it from inflammation "it should be thrust." This poor marty, capped, bled and labeled by as many hands and in as many ways, as the senile land turtle, whose first inscription portrays the hand of Noah of the Ark, too often returns death for our treatment and baffles our efforts to account for all its varied functions. It consists of an internal portion of highly organized, structural membrane, and an external coating. which, though thin, is very dense, and in all probability is a modified form or higher grade of delicate connective tissue and conforms in appearance to the basement membrane of the glands and kidneys. Nerve fibres and capillaries enter into its structure, and, from continued inflammation,.it becomes hypertrophied, and to this condition we ascribe the primary cause of the phenomenon of calcification of the tooth pulp.

Dr. Beale, in speaking of basement membrane, says, "it has no visible holes or pores, but it is readily permeable to fluids, in many cases it permits the trans-udation of fluids in both directions, in some instances only in one. Sometimes it allows one fluid to pass in one direction and another in the opposite."

The most interesting and remarkable substance yet observed is the fluid or bioplasm, which may be expressed from the pulp, and a very similar one in which it is imbedded in its normal abode. The appearance of this fluid from the pulp is not unlike the liquor sanguinus of the blood. It contains what would first appear to be blood corpuscles, but, by the addition of reagents, we find them to be much smaller and granular, ragged and stellate in form. In color also they vary—some are grey, others dark. These pulp granules have plenty of room to float in their liquor without impingement, but not so of those in the solution which lies around and between the pulp and the open mouths of the convoluted canaliculi of the dentine, and which enter these convolutions, carrying the pabulum in the shape of millions of granular bioplasts which germinated in the blood, were nurtured in the pulp and passed by exosmosis, clothed with new germinal, formative and life giving power of the most remarkable kind, through the basement membrane into the mountains of dentine and enamel. And it were well that he who mines into this most wonderful of Nature's products should study well the histology thereof.—*Proceedings Ohio Dental Society.*

